# Fertility and parenthood in patients with acute promyelocytic leukemia treated with Arsenic Trioxide and All-Trans retinoic acid

**DOI:** 10.1038/s41408-024-00984-1

**Published:** 2024-01-19

**Authors:** Charanpreet Singh, Manpreet Saini, Arihant Jain, Deepesh Lad, Gaurav Prakash, Alka Khadwal, Shano Naseem, Pankaj Malhotra

**Affiliations:** 1 Department of Clinical Hematology and Medical Oncology, Postgraduate Institute of Medical Education and Research, Chandigarh, India; 2 Department of Hematology, Postgraduate Institute of Medical Education and Research, Chandigarh, India

**Keywords:** Acute myeloid leukaemia, Quality of life

Dear Editor,

Acute Promyelocytic Leukemia (APL) is associated with excellent long-term outcomes with treatment of Arsenic Trioxide (ATO) and All-trans Retinoic Acid (ATRA). Early thrombo-hemorhhagic complications remain the major cause of mortality and most patients who complete induction therapy have long-lasting remissions with a minimal chance of relapse [1, 2]. With the advances in front-line therapy in the treatment of APL, there is now a greater interest towards potential long-term side-effects associated with therapy. In particular, the impact of treatment on long-term fertility remains unclear.

There are contradicting reports in published literature with regards to the impact of Arsenic on fertility. Arsenic has been shown to effect spermatogenesis and cause ovotoxicity in multiple mouse models [3, 4]. There is also some human epidemiological data linking increased Arsenic levels in the body with impaired fertility in both males and females [5, 6]. However, 2 reports looking at the impact on fertility in patients with APL treated with ATO did not find a decrease in fertility in their patients [7, 8]. We looked to study the impact on fertility of patients with APL treated at our center over the past 17 years.

This was a retrospective study conducted at a tertiary care institute in North India. All patients with classical APL treated at our center between 2006-2022 were screened for inclusion in the study. Patients between the age of 18 and 40 years at presentation, who had completed therapy, were in molecular remission and on regular follow-up were included in the study. Patients between the ages of 18 and 40 were selected as that represents the major reproductive time period for most individuals. Patients who had a variant APL or had received anthracyclines for treatment were excluded. Patients who were unmarried or below the age of 18 years were excluded keeping in mind cultural norms and that the minimum age for marriage in India was 18 years during the study period.

The treatment protocol for patients has already been published [1, 9]. In brief, all APL patients received induction with ATO and ATRA. After attaining morphological remission, patients received 3 cycles of consolidation with ATO and ATRA, each 1 month apart. Intermediate and high-risk patients further received a year of maintenance therapy with ATRA, 6-mercaptopurine and oral weekly methotrexate.

For the study, patients fulfilling the inclusion criteria were interviewed telephonically and details regarding any pregnancies post-therapy completion were noted. Data regarding any adverse pregnancy outcomes, including pre-term birth, miscarriage and spontaneous abortions was noted. Information was also gathered regarding the use of artificial reproductive techniques for conception, as well as the presence of any physical anomalies in the children born after therapy completion.

Two-hundred Seventy Eight patients with classical APL were treated with ATO and ATRA at our center during the study period. Out of these, 60 patients were between the ages of 18-40 years who had completed therapy, were in molecular remission and on regular follow-up, and were included in the study. There was an equal number of male and female patients (30 each) in the study. The median age of the patients at diagnosis was 23 years (Range 18–33 years). Three patients were unmarried and hence were not questioned further regarding fertility.

We first looked at patients who had not conceived children after therapy completion. Forty six patients (80.7%) did not try to conceive a child post therapy. Most patients cited their family being complete as the main reason for not trying to conceive another child (N = 40; 86.9%). Four patients did not try to conceive a child post therapy due to fear of disease returning, while 1 patient each did not try to conceive a child due to fear of risk to the unborn child and because they had not yet planned for a family.

We then looked at the patients who had conceived children post therapy (Table 1). Eleven patients (19.3%) had 12 children post therapy, including 7 male and 4 female patients. Ten of these 11 patients had intermediate or high-risk APL and had also received 6 mercaptopurine and oral weekly methotrexate for 1 year as maintenance therapy. The median time from therapy completion to first successful conception was 1.5 years (Range 0.5–3 years). Seven of these eleven patients did not have children prior to the diagnosis and therapy for APL. The median cumulative ATO dose during therapy was 996 mg (Range 648 mg–1188 mg). All patients who tried to conceive a child were able successfully conceive and have a successful pregnancy post treatment for APL. None of the patients used any fertility therapy for conception of pregnancy. There was no history of pre-term birth, miscarriages or spontaneous abortions in any patient. None of the children had any obvious congenital abnormalities as reported by the patients.

**Table 1:** Details of the patients who conceived children post therapy completion for APL.

Patient details	Gender	Age at diagnosis (in years)	Time from therapy completion to parenthood (in years)	Cumulative ATO dose	APML Risk Class
P1	Female	19	1	840 mg	Intermediate
P2	Female	30	0.5	896 mg	Low
P3	Male	21	1.5	1188 mg	Intermediate
P4	Male	23	1.5	784 mg	Intermediate
P5	Male	24	2	1120 mg	Intermediate
P6	Female	23	1.5	1120 mg	High
P7	Female	29	1	684 mg	High
P8	Male	23	1.5	996 mg	Intermediate
P9	Male	18	1	728 mg	Intermediate
P9	Male	18	3	728 mg	Intermediate
P10	Male	33	1.5	1134 mg	High
P11	Male	24	1.5	1080 mg	Intermediate

All patients, both male and female, desirous of a pregnancy/child post completion of therapy for APL were able to do so without use of fertility therapy and no apparent congenital abnormalities were seen in the children in our study.

Studies done on mouse models have elucidated the effect of Arsenic on the male and female reproductive system. Arsenic trioxide has been shown to effect the autophagy system and cause an imbalance in the oxidant-antioxidant system through inhibition which leads to ovarian dysfunction [4]. Similarly, Arsenic Trioxide has also been shown to reduce spermatozoa number and alter the structure of the Leydig cells in mice models [3]. Epidemiological data from Bangladesh linked increased levels of arsenic in groundwater with a higher incidence of stillbirth, spontaneous abortion and neonatal death [5]. Similarly, in a study from China, seminal plasma Arsenic levels were associated with decreased sperm concentration and alpha-glucosidase activity [6]. However, similar to our study, data from patients with APL treated with ATO do not reveal a decrease in fertility [7, 8]. This difference may be due to the fact that intra-venous ATO has a half life of 12 ± 3 h and hence, levels in the human body would be minimal 6–12 months post-therapy completion and would therefore, have a minimal impact on fertility [10]. Further, the median ATO dose received by the patients is higher than the dose mentioned in the paper by Gupta et al, negating that a higher dose of ATO could still impact human APL patients in terms of fertility.

Another interesting aspect of our study is the reasons many patients did not try to conceive children post-therapy completion. While the most common reason was that they had completed their families, 5 patients did not try to conceive due to fear- either of the disease coming back or of possible disease in the child. This highlights the need for proper counselling of the patient even on follow-up so that they may not refrain from parenthood due to unwarranted fears.

Our study has some limitations. The study was conducted as a telephonic interview of patients, and hence many documents regarding period of gestation as well as baby weight were either unavailable or could not be verified. A detailed assessment of the children born after therapy completion was not done, so internal congenital abnormalities may have been missed till they manifest later in life. Further, due to the small sample size, specific differences in male and female fertility after ATO exposure cant be elucidated.

## Data Availability

The data will be made available on reasonable request by contacting the corresponding author.
